# Anti-aging effects of coffee

**DOI:** 10.18632/aging.101287

**Published:** 2017-08-29

**Authors:** Keita Takahashi, Akihito Ishigami

**Affiliations:** Molecular Regulation of Aging, Tokyo Metropolitan Institute of Gerontology, Tokyo 173-0015, Japan

**Keywords:** aging, mTOR, coffee, caffeine

There are numerous habitual coffee drinkers in the world, and elderly people are no exception. Recently, coffee has been recognized as an effective beverage for healthful aging, especially with respect to maladies such as cardiovascular disease [1] and mild cognitive impairment [2]. Moreover, several human studies have revealed that habitual coffee intake reduces the all-cause mortality in Japanese and several other population groups [3,4] and mortality from heart disease and cerebrovascular disease [3]. Coffee contains caffeine and many kinds of polyphenols. Caffeine has several effects on aging, especially through inhibiting the mammalian target of rapamycin (mTOR) complex 1 (mTORC1) and prolonging the life span of fission yeast [5]. Moreover, the polyphenol chlorogenic acid has many beneficial effects, e.g., lowering fat accumulation in diet-induced obese mice by downregulating sterol regulatory element-binding protein 1 [6]. These studies indicate that one of the most consumed beverages, coffee, has potential anti-aging effects that contribute to the prevention of age-related diseases. However, the mechanisms and effects of coffee are not fully understood with respect to aging or age-related diseases.

**Figure 1:**
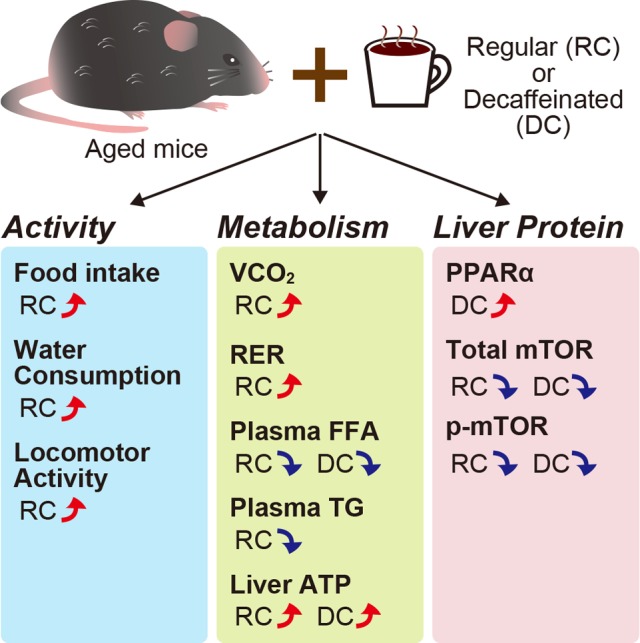
Effects of coffee consumption on aged mice Coffee consumption in aged mice reduced the mTOR and p-mTOR levels in the liver. mTOR, mammalian target of rapamycin; PPARα, peroxisome proliferator-activated receptor α; RER, respiration exchange ratio; VCO_2_, volume of carbon dioxide excretion.

Recently, we elucidated the effects of caffeine-containing regular coffee and decaffeinated coffee consumption on aged mice (Fig. 1) [7]. Regular coffee consumption increased the nocturnal activity of aged mice, including their food intake, water consumption, and locomotor activity, without disrupting the circadian rhythm. We observed no body, liver, or adipose tissue weight changes among all groups during the experimental period. However, we found that regular coffee consumption increased the energy expenditure estimated from CO_2_ excretion and the respiration exchange ratio. To investigate what was excreted in aged mice that consumed coffee, we carried out biochemical and biomolecular analyses. As a result, both regular and decaffeinated coffee consumption were found to reduce free fatty acid levels in the plasma of aged mice. Additionally, both regular and decaffeinated coffee intake increased ATP levels in the liver of aged mice. Protein analyses by western blotting revealed that decaffeinated coffee increased protein levels of peroxisome proliferator-activated receptor (PPAR) α, which is involved in lipid β-oxidation, when compared with the control mice. Interestingly, the total and phosphorylated (Ser2448) mTOR levels in the liver were decreased by consuming coffee containing caffeine or not, though protein and phosphorylated levels of Akt and AMP-activated protein kinase (AMPK), which activate and inhibit mTOR, respectively, were not altered by drinking coffee. Phosphorylated-mTOR (Ser 2448) is an indicator of mTOR complex 1, which is involved in many pathways influencing aging and age-related diseases [8]. This study had no more insight into why or how coffee intake reduced the mTOR and p-mTOR levels in the liver; however, these results suggested that both regular and decaffeinated coffee consumption have effects on aging and age-related diseases such as cancer by decreasing mTOR [8]. Furthermore, as mentioned above, coffee consumption has effects on mortality [3,4]; our study provides more information about the effects of regular coffee-consumption on not only lifespan but also healthfulness by increasing activity and decreasing free fatty acid in the bloodstream.

Further analysis of coffee's relationship with the mTOR regulating pathway and a healthy lifespan may be a breakthrough in the therapy of age-related disease using coffee. Our study provided several new indications about coffee consumption on aged people so that a future study can provide more potential insights about coffee as a health food.
